# PTBS bei RettungssanitäterInnen: eine „Wie-Berufskrankheit“

**DOI:** 10.1007/s00115-023-01539-8

**Published:** 2023-09-27

**Authors:** Harald Dreßing, Wolfgang Spellbrink, Andreas Hoell

**Affiliations:** 1 Zentralinstitut für Seelische Gesundheit Mannheim, Medizinische Fakultät Mannheim, Universität Heidelberg, J 5, 68159 Mannheim, Deutschland; 2 Bundessozialgericht, Graf-Bernadotte Platz 5, 34119 Kassel, Deutschland

## Rechtliche Rahmenbedingungen

Berufskrankheiten sind nach § 9 SGB VII solche Krankheiten, die nach den Erkenntnissen der medizinischen Wissenschaft durch besondere Einwirkungen verursacht sind, denen bestimmte Personengruppen durch ihre versicherte Tätigkeit in erheblich höheren Grade als die übrige Bevölkerung ausgesetzt sind [1]. ArbeitnehmerInnen können von einem Unfallversicherungsträger jedoch nur dann Leistungen verlangen, wenn eine Krankheit ausdrücklich in den Anhang der Berufskrankheiten-Verordnung aufgenommen wurde (sog. „Listen-BK“). Bisher ist keine psychische Störung in dieser Liste aufgeführt. Sofern neue wissenschaftliche Erkenntnisse vorliegen, kann der vom BMAS berufene „Ärztliche Sachverständigenbeirat für Berufskrankheiten“ empfehlen, eine Krankheit in diese Liste aufnehmen. Auch wenn eine solche Empfehlung vorliegt, vergehen bis zur tatsächlichen Umsetzung oft einige Jahre. Deshalb müssen die Berufsgenossenschaften neue Krankheiten als „Wie-Berufskrankheit“ nach § 9 Abs. 2 SGB VII anerkennen, wenn sie nach neuen Erkenntnissen der medizinischen Wissenschaft die Voraussetzungen für eine Bezeichnung als Berufskrankheit erfüllen. Psychische Störungen wurden bislang auch nicht als „Wie-Berufskrankheit“ anerkannt, weil der ÄSVB davon ausging, dass bei diesen Erkrankungen keine gesicherten wissenschaftlichen Erkenntnisse für eine Anerkennung vorliegen, wobei sich der ÄSVB dabei insbesondere auf eine Veröffentlichung von Bolm-Audorff stützte [2]. Die Sozialgerichte folgten bislang in ihrer Entscheidungspraxis dieser Einschätzung des ÄSVB und lehnten die Anerkennung der posttraumatischen Belastungsstörung (PTBS) im Allgemeinen und bei RettungssanitäterInnen im Besonderen als eine „Wie-Berufskrankheit“ ab. Das Bundessozialgericht ist nun erstmals nicht der Einschätzung des ÄSVB gefolgt und hat selbst ein Gutachten bei dem Autor (HD) in Auftrag gegeben, das sich u. a. auf eine neue ausführliche Metaanalyse stützt, die mit dem Mitautor dieses Artikels (AH) durchgeführt wurde [3]. Das Bundessozialgericht hat daraufhin durch Urteil vom 22.06.2023 die posttraumatische Belastungsstörung bei RettungssanitäterInnen als „Wie-Berufskrankheit“ anerkannt (B 2 U 11/20 R). Damit wurde erstmals eine psychische Störung von der Rechtsprechung als „Wie-Berufskrankheit“ anerkannt.

## Epidemiologische Datenlage

Die internationale Forschung hinsichtlich eines Zusammenhangs zwischen einer Berufstätigkeit als RettungssanitäterIn und der Entstehung einer posttraumatischen Belastungsstörung steht nicht im Zentrum der Forschungsaktivitäten. Bisherige Studien haben mit unterschiedlichen Methoden PTBS-Prävalenzraten bei RettungssanitäterInnen ermittelt, die sehr heterogen sind und in einer Spanne von 0–46 % liegen [4, 5]. Bei der Interpretation dieser Zahlen sind methodische Limitationen zu beachten wie z. B. die unterschiedliche Auswahl der Untersuchungsinstrumente, heterogene Ein- und Ausschlusskriterien und unterschiedliche Definitionen der PTBS in den jeweiligen Diagnose- und Klassifizierungssystemen psychischer Störungen. Die große Mehrzahl aller bisher vorliegenden Publikationen zu der Fragestellung kommt allerdings zu einer deutlich höheren PTBS-Prävalenz in der Gruppe der RettungssanitäterInnen im Vergleich zur Allgemeinbevölkerung. In der von den Autoren dieser Arbeit (HD und AH) durchgeführten aktuellen Metaanalyse wurde nicht nur die 12-Monats-PTBS-Prävalenz bei RettungssanitäterInnen mit der der Allgemeinbevölkerung im arbeitsfähigen Alter verglichen, sondern es wurden auch Personen, die von Naturkatastrophen und von Menschen verursachten Katastrophen betroffen waren, als Vergleichsgruppen herangezogen. Dabei fand sich bei den RettungssanitäterInnen eine 12-Monats-PTBS-Prävalenz von 20,02 % (95 %iges Konfidenzintervall: 16,09–26 %), in der Allgemeinbevölkerung von 3,06% (2,31–3,90 %), in der Gruppe der von einer Naturkatastrophe betroffenen Personen von 15,59 % (11,93–19,63 %) und in der von einer menschlich verursachten Katastrophe von 12,05 % (9,17–15,20 %; [3]). Die 12-Monats-PTBS-Prävalenzen der RettungssanitäterInnen sind also erheblich höher, selbst bei einem Vergleich mit Personengruppen, die einem erheblichen Trauma ausgesetzt waren. Ein weiterer bedeutsamer Befund dieser Metaanalyse ergab sich aus einem Vergleich der PTBS-Prävalenz bei RettungssanitäterInnen, bei denen eine Exposition mit einem definierten katastrophalen traumatischen Ereignis festgestellt wurde, mit der PTBS-Prävalenz von RettungssanitäterInnen, für die keine Exposition mit einem solchen besonderen Ereignis vorlag, sondern nur übliche kumulative Traumatisierungen durch wiederholtes Erleben traumatisierender Ereignisse wie z. B. der wiederholten Konfrontation mit aversiven Details. In der PTBS-Definition des DSM 5 ist diese Form der Traumaerfahrung explizit als hinreichendes Traumaeingangskriterium erwähnt. Die Subgruppenanalyse ergab, dass RettungssanitäterInnen, die „nur“ üblichen kumulativen Traumatisierungen durch das wiederholte Erleben traumatisierender Ereignisse während der Arbeit exponiert waren, sogar eine um durchschnittlich 8,0 % höhere PTBS-Prävalenz hatten als die Vergleichsgruppe der Rettungssanitäter, die bei einem definierten katastrophalen Ereignis eingesetzt waren (23,2 % vs. 15,2 %). Dieser Befund unterstreicht die von Lee und Kollegen [6] hervorgehobene Bedeutung kumulativer niedrigschwelliger traumatischer Erlebnisse und könnte für ein besonderes berufliches Risiko von RettungssanitäterInnen sprechen, eine PTBS zu entwickeln.

## Gutachtliche Einordnung

Welche rechtlich-normativen Schlussfolgerungen aus feststellbaren signifikanten Unterschieden in der PTBS-Prävalenz bei bestimmten Berufsgruppen im Hinblick auf die Annahme einer „Wie-Berufskrankheit“ zu ziehen sind, obliegt nicht der medizinischen Expertise, sondern ist Gegenstand der rechtlich-normativen Würdigung [1]. Dabei ist zu beachten, dass der Vergleich zwischen Prävalenzzahlen für psychische Störungen bei RettungssanitäterInnen und den Prävalenzen in der Allgemeinbevölkerung vor der methodischen Limitation steht, dass die eingesetzten Studiendesigns und Untersuchungsinstrumente oft verschieden sind. Nur äußerst wenige Studien berücksichtigen vergleichbare Stichproben mit Personen der Allgemeinbevölkerung, gleiche Untersuchungsinstrumente und ein rigoroses Matching zwischen den Stichproben. Im Hinblick auf die Fragestellung, ob für eine bestimmte Erkrankung ein berufsspezifisches Risiko anzunehmen ist, wird teilweise ein mindestens zweifach erhöhtes gruppenspezifisches Risiko gefordert, an einer bestimmten Krankheit zu erkranken [7], wobei dieses sog. „Verdoppelungsrisiko“ allerdings nicht im Gesetz steht und daher kein zwingendes normatives Kriterium darstellt [1]. Unabhängig von dieser juristischen Frage ist aber ein gruppenspezifisches Verdopplungsrisiko für die PTBS bei Rettungssanitätern auf der Basis der vorliegenden Studien zu bejahen. Zu betonen ist, dass die Studien zwar eine signifikante Assoziation zwischen beruflicher Tätigkeit und dem Risiko, an einer PTBS zu erkranken, aufzeigen, aufgrund der angewandten Methodik aber keine Kausalität nachweisen können. Hierzu fehlt es national wie international an prospektiven Studien mit einem Fall-Kontrollgruppen-Design. Die von Autoren dieser Arbeit (HD und AH) durchgeführte Metaanalyse [3] untermauert aber die von Flatten und Kollegen vertretene Auffassung, dass vorliegende Befunde zur Bedeutsamkeit kumulativer Belastungserfahrungen sowie Erkenntnisse aus der neurobiologischen Forschung die Anerkennung einer PTBS als Berufskrankheit u. a. bei der Gruppe der RettungsassistentInnen rechtfertigen [8]. Diese Auffassung hat das Bundessozialgericht in seinem Urteil nun für die Berufsgruppe der RettungssanitäterInnen bestätigt (2 U 11/20 R).

## Fazit für die Praxis

- Die Anerkennung der PTBS als „Wie-Berufskrankheit“ bei der Berufsgruppe der RettungssanitäterInnen durch das Bundessozialgericht ist ein Meilenstein im Hinblick auf eine geleichberechtigte Behandlung psychischer Störungen und körperlicher Erkrankungen.
- Das Bundessozialgericht hat damit zugleich das Definitionsmonopol des ÄSVB, welche Krankheiten als Berufskrankheiten anzuerkennen sind, infrage gestellt.
- Es ist in Zukunft mit einer größeren Anzahl von Anträgen von RettungssanitäterInnen zu rechnen.
- Hierbei ist ein sorgfältiges Vorgehen bei der Begutachtung der Antragsteller unabdingbar und sehr gründlich zu prüfen und gutachtlich auf der Befundebene zu belegen, dass die diagnostischen Kriterien der PTBS auch tatsächlich vorliegen.
